# The importance of common and the irrelevance of rare species for partition the variation of community matrix: implications for sampling and conservation

**DOI:** 10.1038/s41598-020-76833-5

**Published:** 2020-11-13

**Authors:** Leandro Schlemmer Brasil, Thiago Bernardi Vieira, André Felipe Alves Andrade, Rafael Costa Bastos, Luciano Fogaça de Assis Montag, Leandro Juen

**Affiliations:** 1 Programa de Pós-Graduação em Zoologia, Universidade Federal do Pará, Belém, Pará Brasil; 2 Laboratório de Ecologia e Conservação, Universidade Federal do Pará, Belém, Pará Brasil; 3 Programa de Pós-Graduação em Ecologia e Conservação, Universidade Federal do Pará, Belém, Pará Brasil; 4 Theory, Metacommunity and Landscape Ecology Lab, Universidade Federal de Goias, Goiânia, Goias Brasil

**Keywords:** Ecology, Zoology, Limnology

## Abstract

In community ecology, it is important to understand the distribution of communities along environmental and spatial gradients. However, it is common for the residuals of models investigating those relationships to be very high (> 50%). It is believed that species’ intrinsic characteristics such as rarity can contribute to large residuals. The objective of this study is to test the relationship among communities and environmental and spatial predictors by evaluating the relative contribution of common and rare species to the explanatory power of models. Our hypothesis is that the residual of partition the variation of community matrix (varpart) models will decrease as rare species get removed. We used several environmental variables and spatial filters as varpart model predictors of fish and Zygoptera (Insecta: Odonata) communities in 109 and 141 Amazonian streams, respectively. We built a repetition structure, in which we gradually removed common and rare species independently. After the repetitions and removal of species, our hypothesis was not corroborated. In all scenarios, removing up to 50% of rare species did not reduce model residuals. Common species are important and rare species are irrelevant for understanding the relationships among communities and environmental and spatial gradients using varpart. Therefore, our findings suggest that studies using varpart with single sampling events that do not detect rare species can efficiently assess general distributional patterns of communities along environmental and spatial gradients. However, when the objectives concern conservation of biodiversity and functional diversity, rare species must be carefully assessed by other complementary methods, since they are not well represented in varpart models.

## Introduction

Understanding the distribution of biological communities along environmental and spatial gradients has had several theoretical advances such as the Niche concept^1^, Neutral Theory^2^, and the Meta communities synthesis^3^. Parallel to these advances, the development of methods that quantify the importance of spatial and environmental filters as community predictors^4–6^ have been essential for interpreting the distribution patterns of species in communities^7^.

Niche theory^1^, that predicts species are distributed according to the environmental conditions^8^ and biotic interactions^9^, which determine their distribution along the environmental gradient. Thus, species distribution is solely explained by the conditions and resources present in the studied sites. In contrast, Neutral theory posits that species distribution does not depend of environmental conditions or resources, and that spatially closer sites would have similar communities^10^ due to historical processes such as vicariance and dispersal^2^. Therefore, according to niche theory predictions, the main filters for species distributions are the relationship between species and their environment, along with local extinctions where conditions and resources are inadequate^1^. Alternatively, for Neutral theory, the main filters are species dispersal and the probabilities (random events) of speciation and extinction^2^. Considering these different structuring forces, to understand the distribution patterns of species in a landscape, it is necessary to understand the environmental and spatial mechanisms associated with species^7^.

A way of quantifying the importance of those mechanisms is using variation partitioning models^5^ between environmental and spatial predictors^11^. These models have four elements: the portion explained exclusively by the spatial component, the portion explained exclusively by the environmental component, the portion explained by the interaction between environmental and spatial predictors, and the residual portion, not explained by the model^12^. Though this is a widely accepted method for investigating the effects of niche and neutral mechanisms in community ecology, a recent meta-analysis^7^ revealed it is common for the residual portion of the model to be higher than the explained portion (> 50%)^5^. This was shown in very metacommunities with fish^13^, Odonata^14,15^ and termites^16^ in Amazonian streams, macroinvertebrates in the south^17^ and southeast^18^ of Brazil, and in macrophytes^19^ and beetles^20^ in temperate regions. The high residual variation is associated with environmental or spatial predictors that were not included in the models^5,7^ and/or with a subset of species that have antagonistic responses to the environmental and/or spatial gradients due to autoecological characteristics^21^.

One potential reason for such an antagonistic response is the categorization of species as rare or common, which tend to respond to environmental gradients in an idiosyncratic way^18,22^. Because of that, by analyzing all species in the community together, it is possible to increase the model residual portion. In this context, species that are common and have a large spatial distribution do not have high dispersal limitations and are generalists, possessing environmental plasticity that allows them to survive in different conditions^23^, some of which are considered adverse. While, rare species, on the other hand, have more restricted distributions^24^ and knowledge about their relationship with environmental variables is generally limited. For these reasons, rare species are often not considered in community analyses or have few statistical relevance to community patterns (e.g. in direct ordination analyses)^4^.

To understand the mechanisms and patterns important for community assembly it is essential to create a multitaxon approach, allowing for greaterextrapolation power of the results^25^. Considering studies in stream ecology to jointly evaluate taxa that use different parts of the habitat (e.g.: fish, exclusively aquatic organisms, and Zygoptera adults, which live in the riparian vegetation^13,26^) may provide robust results which could be extrapolated for other organisms of the aquatic biota living in similar habitats.

Thus, our objective is to test the relationship among communities and the environmental and spatial predictors by evaluating the relative contribution of common and rare species to the power of explanation of the models. Our hypothesis is that the residual portion of variation partitioning models will decrease as rare species are removed from the analyses. Rare species would inflate the residual portion as they make the models less parsimonious, since they would add too many species that are not relevant in the relationship among community patterns and spatial and environmental patterns.

## Results

**Figure 1:**
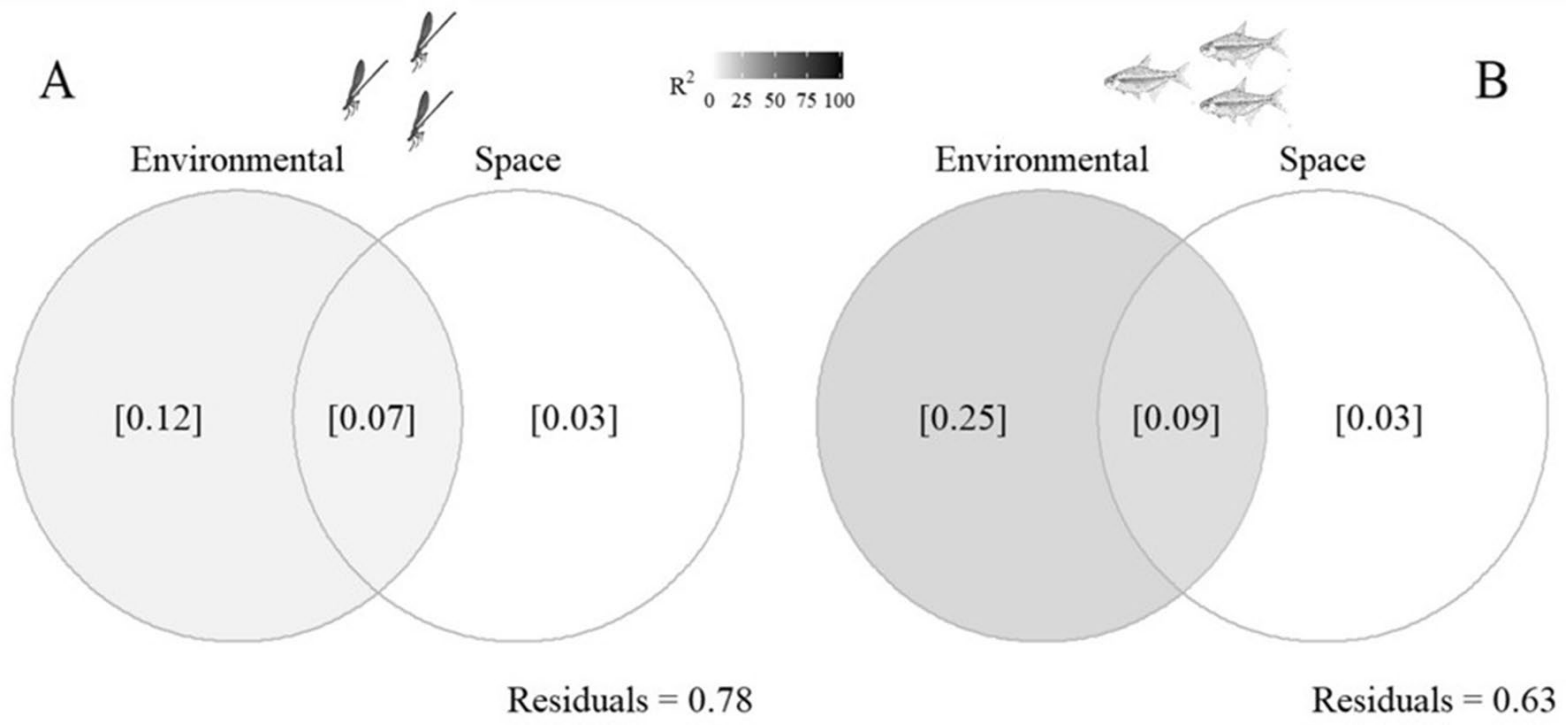
Variation partitioning (pRDA) analyzing the effects of environmental predictors, spatial predictors, the interaction term between environmental and spatial predictors, and the residual portion on all species of Zygoptera and fish communities.

Environmental and spatial predictors explained together 37% and 22% of the variation for Zygoptera and fish, respectively. For fish (25%) and Zygoptera (12%), most of the explanation was for environmental predictors. The spatial component explained only 3% for fish and Zygoptera (Fig. 1).

**Figure 2:**
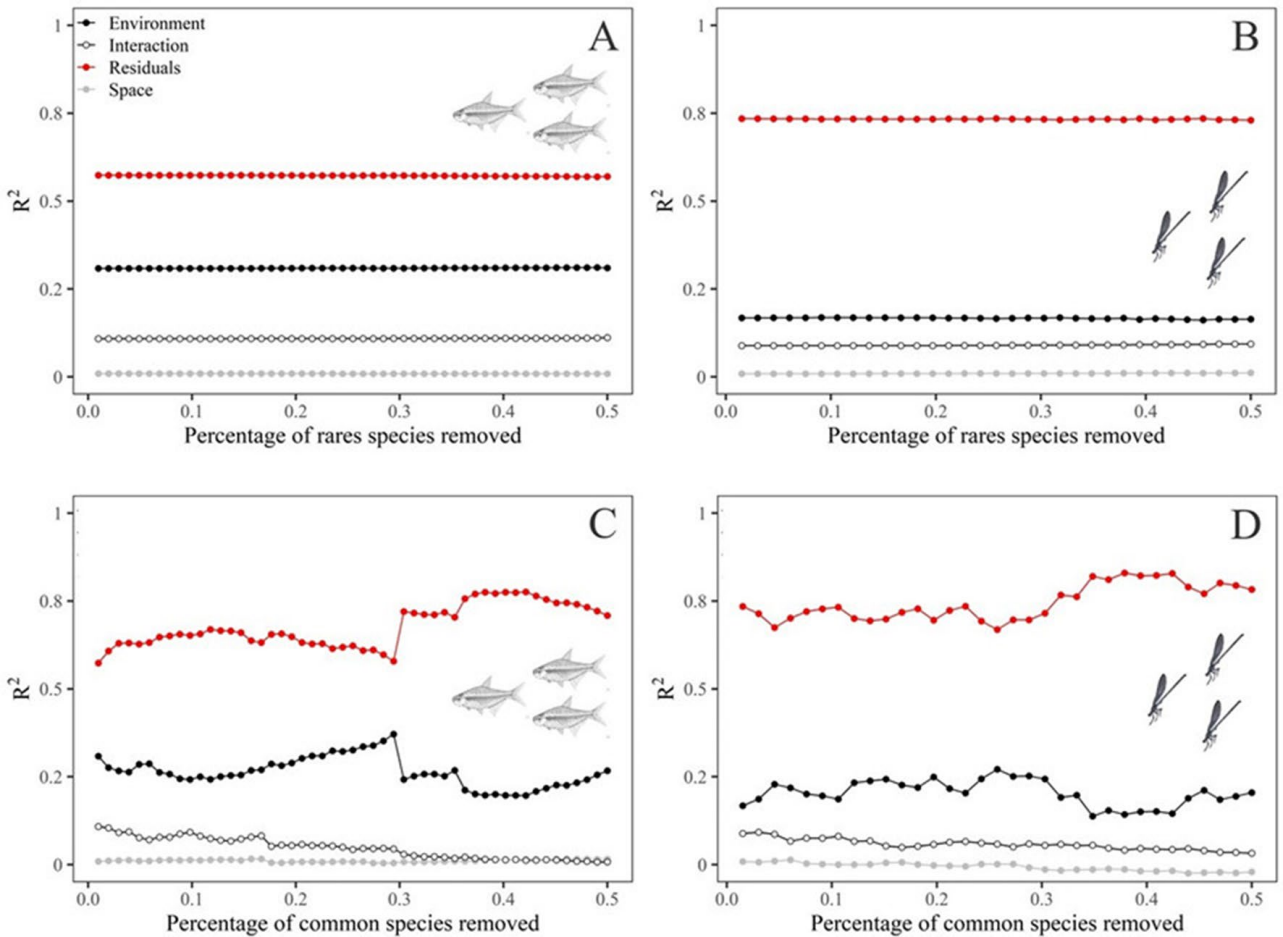
Correlogram showing the R^2^ values of the variation partitioning (pRDA) analyzing the effects of environmental predictors (black circles), spatial predictors (empty circles), the interaction termbetween environmental and spatial (grey circles), and the residual portion (red circles) on fish and Zygoptera communities. A = fish communities with abundance data gradually removing rare species. B = Zygoptera communities with abundance data gradually removing rare species. C = fish communities with presence-absence data gradually removing rare species. D = Zygoptera communities with abundance data gradually removing common species.

The gradual removal of rare species (up to 50% of total community richness) had little effect on the percentage of variation explained by environmental and spatial predictors for Zygoptera (ΔR^2^ = − 0.004, p = 0.053) and fish (ΔR^2^ = − 0.003, p = 0.060) communities. However, the gradual removal of common species (up to 50% of total community richness) strongly altered the percentage of variation explainedby environmental and spatial predictors of fish (ΔR^2^ = 0.135, p = 0.002) communities, but did not significantly differ from the alteration in the explanation of Zygoptera (ΔR^2^ = 0.048, p = 0.536) communities when species are removed at random when species are removed at random (Fig. 2A:D).

## Discussion

**Figure 3:**
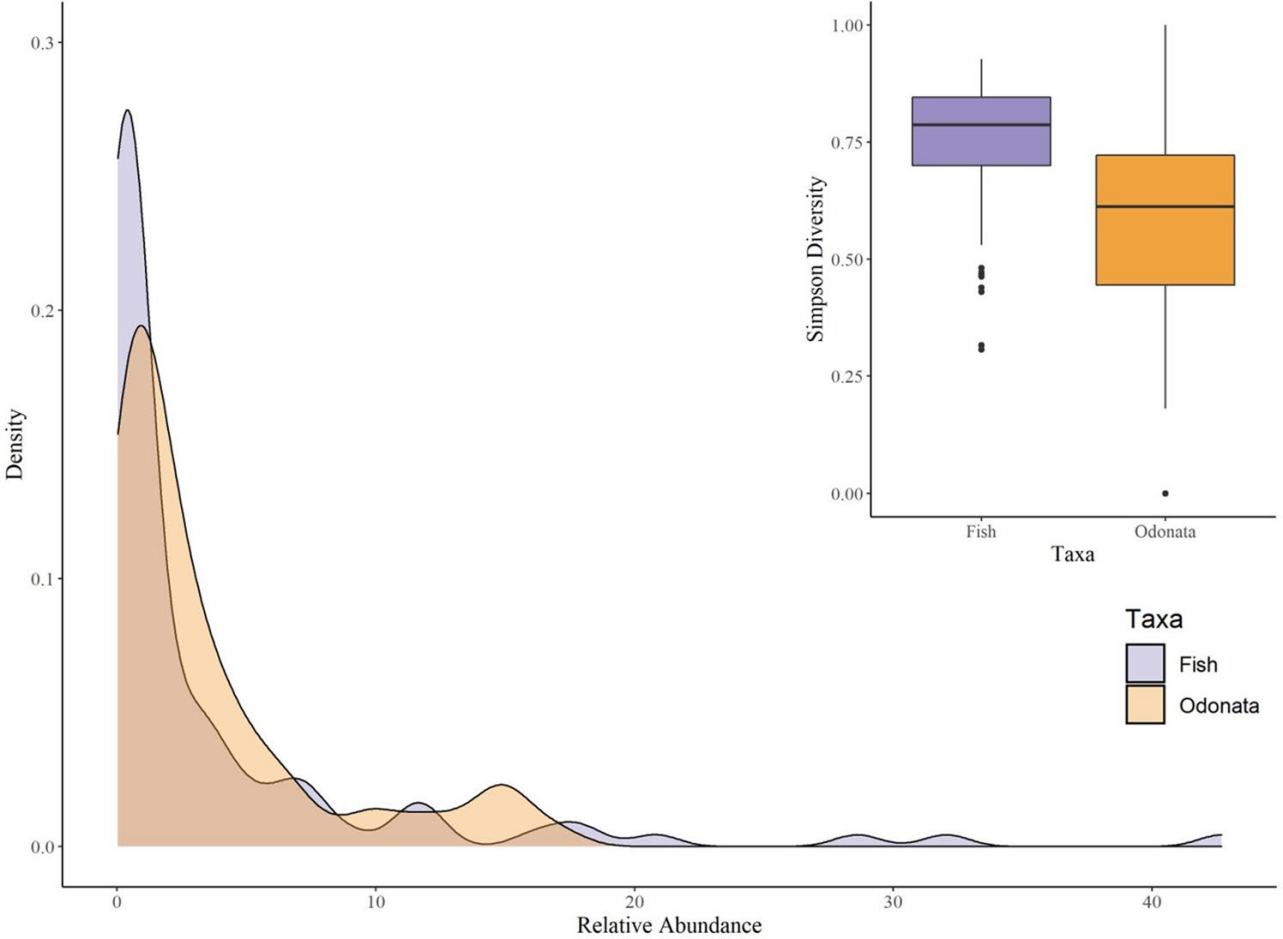
Abundance curves of fish and Odonata communities observed. The boxplot represents the variation of dominance represented by the Simpson index in the subsamples.

Our hypothesis that the residual portion of the variation partitioning models would decrease as rare species were removed was not corroborated. In all scenarios, for both fish and Zygoptera, removing up to 50% of rare species did not reduce residuals in a significant way. However, for the most common species, the removal of only one species had notable effects in the residual portion of the models. This effect is more pronounced in communities in which a few species dominate the community, such as the fish community in our study (Fig. 3), since the removal of the most abundant species steeply reduces the percentage of environmental and spatial variation explained.

Therefore, when analyzing general patterns of community distribution in spatial or environmental gradients using variation partitioning, the exclusion of rare species does not promote significant residual increase. Due to potential analytical problems associated with rare species in community analyses, for example, the great number of zeros in species matrices^11^ and having more species than samples^27^, it is recommended to exclude rare species before performing data analyses. Our results suggest this is possible without harming the visualization of general patterns of communities along spatial and environmental gradients^28^.

In tropical countries, where species diversity is high^29^, big knowledge shortfalls regarding biodiversity distribution^30^ (known as the Wallacean shortfall^31^) exist. Additional financial crisis may reduce research investments^32^, so it is common for the biological sample to be from one sole sampling/field trip^13,26,33,34^. These sampling strategies can result in a low representation of rare species, and, consequently, can affect the detection of diversity patterns^35^. However, despite these possible limitations, our results demonstrate that even if a considerable portion of rare species is not captured this does not make it impossible to evaluate the general distributional patterns of communities in spatial and environmental gradients.

Nonetheless, for conservation^24^ or to assess the functional role of species in ecosystems^36,37^, the exclusion of rare species can bias the results. Functionally, rare species contribute considerably to patterns of richness, specialization, and functional originality of communities^36,37^. Conservation assessment at the community level can disregard rare and important taxa, such as endemic species^38^. Thus, conservation measures are commonly evaluated and proposed at the population level^39^.

**Figure 4:**
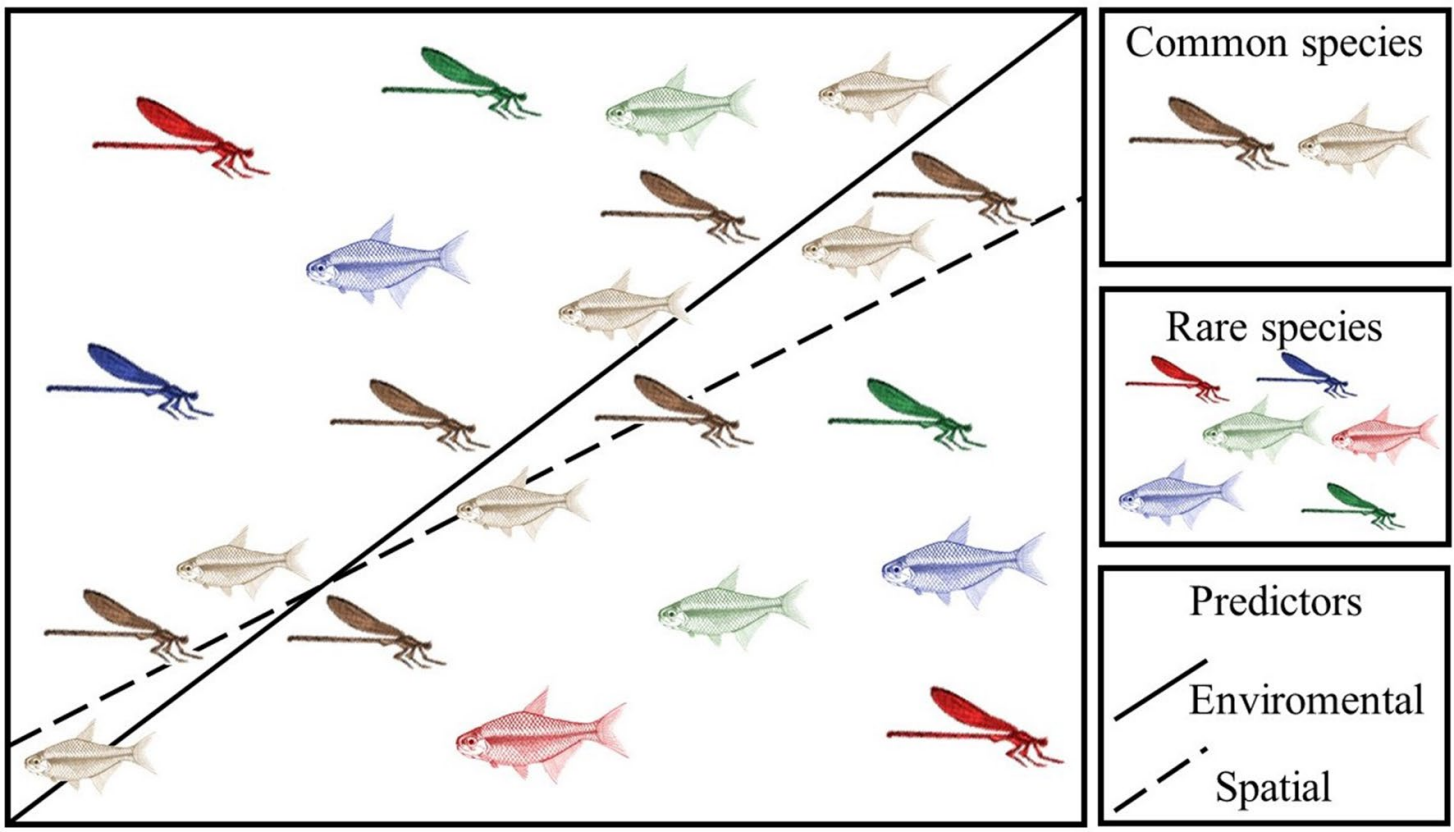
Graphic summary of the main results.

From our findings, we conclude that common species are important, and rare species contribute little for understanding the relationships of communities with spatial and environmental gradients using variation partitioning (Fig. 4). However, when considering other objectives such as conservation or the analysis of functional patterns, information about rare species continues to be essential. We further emphasize that studies with sole sample events often may not be effective in detecting rare species but are efficient in identifying general community distribution patterns along environmental and spatial gradients. Therefore, when necessary, the exclusion of rare species does not harm the interpretation of community patterns in those circumstances.

## Material and methods

### Study area

We sampled Zygoptera and Fish assemblages of small streams (up to 4 m width and a 0.8 m mean depth) in Eastern Amazon (Brazil). We sampled fish in 109 streams and Odonates in 141 streams. The sampling sites are in areas where the natural forest exists but has been suffering with a continuous process of landscape fragmentation due to the advancement of agrosystems (e.g.: agriculture, pasture and logging) in natural areas^13,26^. The study area consists of three macro climatic zones according to the Köppen classification^40^: The Am zone (North), the Aw zone (South), and the Af zone (East). The Am is a tropical rainy climate (also called tropical monsoon climate), Aw is a tropical climate with distinct dry and rainy seasons, and Af is a tropical rainforest climate (also called equatorial climate)^40^.

All methods were carried out in accordance with relevant guidelines and regulations. For Odonata samples the surveys were authorized by the Instituto Brasileiro do Meio Ambiente e dos Recursos Naturais Renováveis (IBAMA, Licence No. 1993421). For Fish samples the Ethics Committee of the Federal University of Pará approved the surveys (CEUA nº 8293020418), and they were conducted under the permission nº 4681-1 granted by the Instituto Chico Mendes de Conservação da Biodiversidade (IcmBIO; Ministério do Meio Ambiente).

### Zygoptera sample

We sampled adults Zygoptera once on each stream. Sampling happened between 2009 and 2018 always between the months of July and December, when the highest diversity of aquatic insects is collected in the Amazon. In each stream, we demarcated a linear transect of 150 m and captured all the Zygoptera adult specimens observed in the span of one hour. We used an entomological net of 40 cm diameter and 65 cm width. To minimize bias related to the different types of Zygoptera thermoregulation (thermal conformers, heliotherms and endotherms), the collection of specimens occurred between 10 a.m. and 2 p.m., when sunlight reaches the stream channel^14,41^.

The specimens were stored according to the protocol proposed by Ref.^42^. We identified the material using taxonomic keys and specialized guidebooks e.g.: Refs.^42–44^, and, when necessary, we sent the material to specialists. The specimens are deposited in the collection of the Zoology Museum of the Federal University of Pará, located in Belém, state of Pará, Brazil.

### Fish sample

The fish were collected in 109 first and third-order streams, along 150 m transects, in the dry season, between July and December of 2012–2015. We selected this period to avoid seasonal variation in the fish assemblage structure^45,46^ and to increase sampling efficiency, which is easier under low streamflow.

The fish were captured using circular 55-cm-diameter dip nets with a 3 mm metallic mesh. This type of dip net is considered an efficient method to collect specimens, allowing the capture of fish in small stream microhabitats, including complex habitats (e.g. riparian vegetation, leaf banks and the inferior portion of wood trunks and branches^47^). The 150 m transect was divided into 10 sections. Each section was sampled by 2 collectors for 18 min, and the total sampling time was 3 h/stream. The specimens were euthanized using anesthetics (Eugenol, following the Brazilian Civil Law nº 11.794/2008), fixed in a 10% formalin solution, and transferred to 70% ethanol after 48 h. In the laboratory, the specimens were identified using taxonomic keys^48,49^ and with the aid of specialists. All specimens were deposited in the ichthyology collection of the Emílio Goeldi Museum (MPEG), Belém, State of Pará, Brazil.

### Environmental predictors

We used environmental variables measured by the protocol of environmental assessment developed by the United States Environmental Protection Agency (US-EPA)^50,51^. We took measurements and observed the characteristics of the habitat in the same 150 m stretch where Zygoptera and fish sampling took place. In total, we obtained 186 measurements: 27 are related to channel morphology and hydraulics, 26 are related to substrate, 28 to organic debris, 60 are related to wood, 16 to the characteristics of the riparian forest, and 29 refer to human influence^13^. We took a series of steps to reduce the number of variables. Initially, we removed variables that had a low (< 40%) coefficient of variation (CV) and the ones that had high values (> 80%). We removed them because predictors that have little variation are not representative in the environmental gradients, and samples with too many zeroes (high coefficient of variation) indicate a sampling problem^13,26^.

Considering this selection criterion for the 186 initial environmental variables, there were 139 remaining predictors for Zygoptera, and 51 for Fish. This difference is because some of the collection sites were distinct for fish and Zygoptera. After that, we used those environmental matrices to perform a model selection (forward stepwise) with species composition (separately for Zygoptera and fish). We used this method of selection to eliminate predictors that did not associate with the species matrices and to avoid inflated residual values because of the use of spurious variables^4^. To avoid multicollinearity in further analyses, we performed Principal Component Analyses (PCA) with the original predictors and used the ordination axes as the predictive variables. To avoid information loss, we retained the axes until the sum of explanation (eigenvalue) was equal to or higher than 90%^52^. For Zygoptera and fish, seven axes were necessary for > 90% in PCA (Supplementary Information).

### Spatial predictors

**Figure 5:**
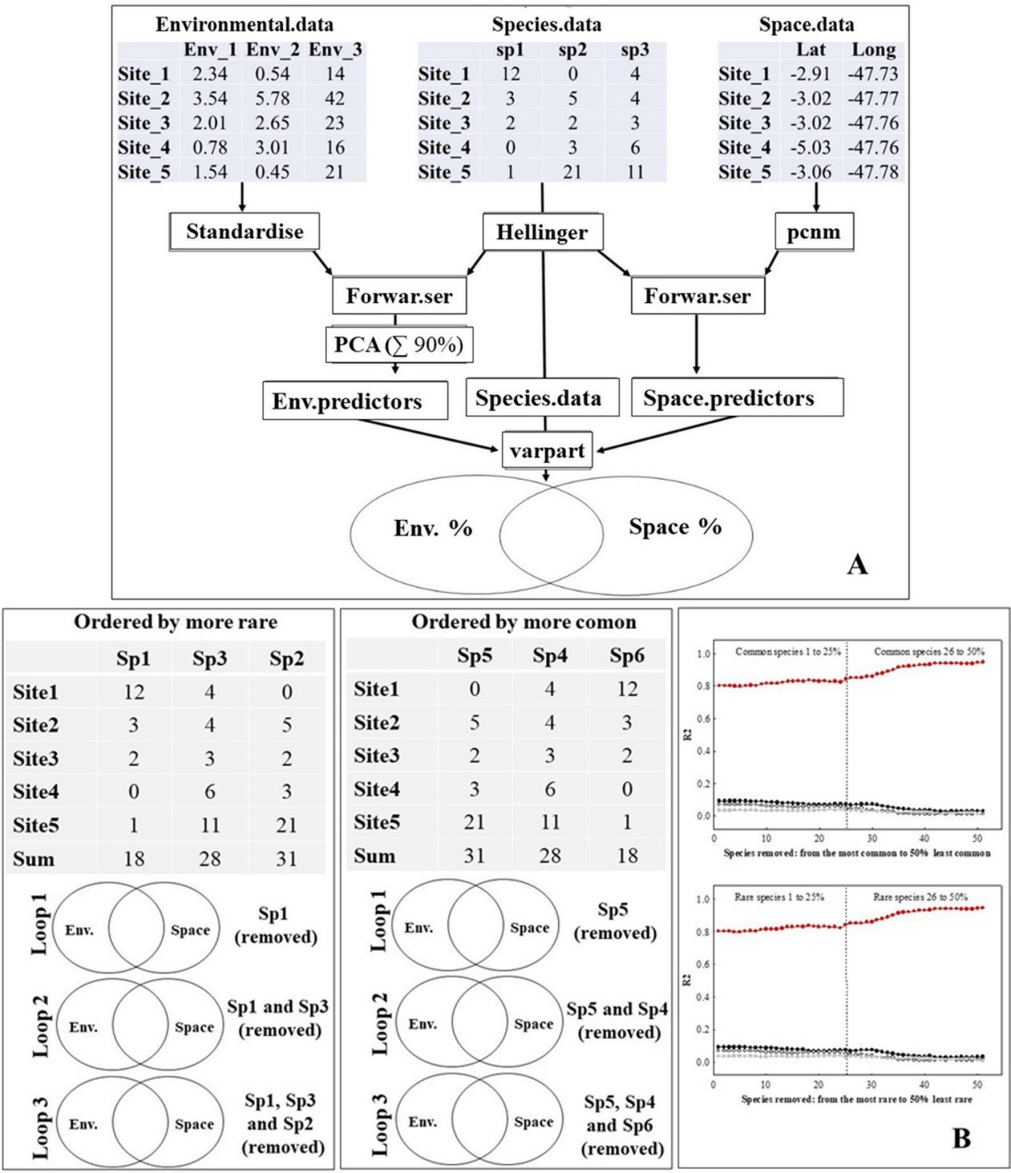
(**A**) The analytic procedure of variation partitioning using environmental and spatial predictors and species composition. The graphic model illustrates the abundance-based species composition data. (**B**) The analytic procedure used to analyze the effects of environmental predictors (black circles), spatial predictors (empty circles), the interaction term between environmental and spatial predictors (grey circles), and the residual portion (red circles), while gradually removing the rarest and most common species. *Env *environmental.

It is expected for communities that are spatially closer to have similar species composition^53^. This happens because there is a higher likelihood of a migratory species flow between environmental patches that are spatially closer^3^. Consequently, when we analyze biological communities through space it is necessary to understand how much of the similarity or dissimilarity among species relates only with the spatial distribution of the sampling sites. To analyze the importance of space, we used spatial filters^6^. For this approach, we used latitude and longitude data of each sampling site to calculate Principal Coordinates of Neighbour Matrices (PCNM). Then, we used the set of vectors as the spatial predictors. To select which vectors are important predictors for the communities, we performed a forward selection model using the PCNMs and the community matrices of Zygoptera and Fish, respectively (Fig. 5A). We used two sets of spatial predictors (PCNMs), one for each taxonomic group. Three PCNMs were selected as spatial predictors for Zygoptera and two for Fish.

### Data analysis

To test the hypothesis that the residual portion of the model would decrease as rare species were removed, we performed the models for each taxonomic group (Zygoptera and Fish). For each taxonomic group, we used the abundance matrix to perform variation partitioning (varpart function from the vegan package in R) using the whole community and the environmental and spatial predictors (Fig. 5A). This was considered the reference value. Then, we built a function where in each loop one species was removed from the community matrix and then the variation partitioning was performed again. The procedure of species removal was executed from the rarest species to the most common species and from the most common species to the rarest species. The species were removed until the resulting richness was equal to 50% of the original community richness. To facilitate interpretation of the results, we created a correlogram using the proportion of variation explained by the environmental, spatial, interaction term, and residual components of each routine step and of each dataset, with the number of species removed from the analysis (Fig. 5B). We tested our hypothesis that residual variance decreases when rare species are removed by contrasting the difference in residual variation before and after removing the rare species (ΔR^2^ = R^2^Before-R^2^After) against a null model in which we took caution to perform a stratified sample of our original community, in order to create subsamples that resemble the distribution of relative abundances in the original community. Species from our subsamples were removed randomly and we calculated the difference in residual variation before and after removing 50% of the community (ΔR^2^). We repeated this procedure 999 times to create a distribution of values for ΔR^2^.We then contrasted our values obtained when removing rare fish and Odonata species against this distribution to obtain a measure of significance for the effect of removing rare species on the proportion of residual variation.

## Supplementary information

Supplementary Information.

## References

[1] Hutchinson GE (1959). Homage to santa rosalia or why are there so many kinds of animals?. Am. Nat..

[2] Hubbell SP (2001). The Unified Neutral Theory of Biodiversity and Biogeography.

[3] Leibold MA, Holyoak M, Mouquet N, Amarasekare P, Chase JM, Hoopes MF, Holt RD, Shurin JB, Law R, Tilman D, Loreau M, Gonzalez A (2004). The metacommunity concept: A framework for multi-scale community ecology. Ecol. Lett..

[4] Legendre P, Legendre L (2012). Numerical Ecology.

[5] Cottenie K (2005). Integrating environmental and spatial processes in ecological community dynamics. Ecol. Lett..

[6] Dray S, Legendre P, Peres-Neto PR (2006). Spatial modelling: a comprehensive framework for principal coordinate analysis of neighbour matrices (PCNM). Ecol. Model..

[7] Leibold MA, Chase JM (2018). Metacommunity Ecology.

[8] Grinnell J (1917). Field tests of theories concerning distributional control. Am. Nat..

[9] Elton C (1946). Competition and the structure of ecological communities. J. Anim. Ecol..

[10] Griffith DA, Peres-Neto PR (2006). Spatial modeling in ecology: The flexibility of eigenfunction spatial analyses. Ecology.

[11] Peres-Neto PR, Legendre P, Dray S, Borcard D (2006). Variation partitioning of species data matrices: Estimation and comparison of fractions. Ecology.

[12] Oksanen, J. *et al*. Vegan: community ecology package. R package version 2.4–5. https://cran.r-project.org/web/packages/vegan/index.html (2017).

[13] Montag LFA, Leão H, Benone NL, Monteiro-Júnior CS, Faria APJ, Nicacio G, Ferreira CP, Garcia DHA, Santos CRM, Pompeu PS, Winemiller KO, Juen L (2019). Contrasting associations between habitat conditions and stream aquatic biodiversity in a forest reserve and its surrounding area in the Eastern Amazon. Hydrobiologia.

[14] Juen L, De Marco P (2011). Odonate biodiversity in terra-firme forest streamlets in Central Amazonia: On the relative effects of neutral and niche drivers at small geographical extents. Insect Conserv. Divers..

[15] Brasil LS, Oliveira-Júnior JM, Calvão LB, Carvalho FG, Monteiro-Júnior CS, Dias-Silva K, Juen L (2018). Spatial, biogeographic and environmental predictors of diversity in Amazonian Zygoptera. Insect Conserv. Divers..

[16] Dambros CS, Morais JW, Azevedo RA, Gotelli NJ (2017). Isolation by distance, not rivers, control the distribution of termite species in the Amazonian rain forest. Ecography.

[17] Hepp LU, Landeiro VL, Melo AS (2012). Experimental assessment of the effects of environmental factors and longitudinal position on alpha and beta diversities of aquatic insects in a neotropical stream. Int. Rev. Hydrobiol..

[18] Siqueira T, Bini LM, Roque FO, Couceiro SRM, Trivinho-Strixino S, Cottenie K (2012). Common and rare species respond to similar niche processes in macroinvertebratemetacommunities. Ecography.

[19] Alahuhta J, Heino J (2013). Spatial extent, regional specificity and metacommunity structuring in lake macrophytes. J. Biogeogr..

[20] Heino J, Alahuhta J (2015). Elements of regional beetle faunas: Faunal variation and compositional breakpoints along climate, land cover and geographical gradients. J. Anim. Ecol..

[21] Algarte VM, Rodrigues L, Landeiro VL, Siqueira T, Bini LM (2014). Variance partitioning of deconstructed periphyton communities: Does the use of biological traits matter?. Hydrobiologia.

[22] Brasil LS, Juen L, Giehl NFS, Cabette HSR (2017). Effect of environmental and temporal factors on patterns of rarity of ephemeroptera in stream of the braziliancerrado. Neotrop. Entomol..

[23] Gaston KJ (2010). Valuing common species. Science.

[24] Gaston KJ (2012). The importance of being rare. Ecology.

[25] Lários MC, Cunha CN, Penha J, Landeiro VL, Pinho JB, Aragona M (2017). Evidence of cross-taxon congruence in Neotropical wetlands: Importance of environmental and spatial factors. Glob. Ecol. Conserv..

[26] Juen L, Cunha EJ, Carvalho FG, Ferreira MC, Begot TO, Andrade AL (2016). Effects of oil palm plantations on the habitat structure and biota of streams in eastern Amazon. River Res. Appl..

[27] Legendre P, Anderson MJ (1999). Distance-based redundancy analysis: Testing multispecies responses in multifactorial ecological experiments. Ecol. Monogr..

[28] Borcard D, Gillet F, Legendre P (2018). Numerical Ecology with R.

[29] Barlow J, França F, Gardner TA, Hicks CC, Lennox GD, Berenguer E (2018). The future of hyperdiverse tropical ecosystems. Nature.

[30] Bini LM, Diniz-Filho JAF, Rangel TF, Bastos RP, Pinto MP (2006). Challenging Wallacean and Linnean shortfalls: Knowledge gradients and conservation planning in a biodiversity hotspot. Divers. Distrib..

[31] Whittaker RJ, Araújo MB, Jepson P, Ladle RJ, Watson JE, Willis KJ (2005). Conservation biogeography: Assessment and prospect. Divers. Distrib..

[32] Crouzeilles R, Feltran-Barbieri R, Ferreira MS, Strassburg BB (2017). Hard times for the Brazilian environment. Nature ecology & evolution..

[33] Vieira TB, Pavanelli CS, Casatti L, Smith WS, Benedito E, Mazzoni R (2018). A multiple hypothesis approach to explain species richness patterns in neotropical stream-dweller fish communities. PLoS ONE.

[34] Brasil LS, Silverio DV, Cabette HSR, Batista JD, Vieira TB, Dias-Silva K, Juen L (2019). Net primary productivity and seasonality of temperature and precipitation are predictors of the species richness of the Damselflies in the Amazon. Basic Appl. Ecol..

[35] Kéry M, Schmid H (2004). Monitoring programs need to take into account imperfect species detectability. Basic Appl. Ecol..

[36] Leitão RP, Zuanon J, Villéger S, Williams SE, Baraloto C, Fortunel C (2016). Rare species contribute disproportionately to the functional structure of species assemblages. Proc. R. Soc. B Biol. Sci..

[37] Pereira DFG, Oliveira-Junior JMB, Juen L (2019). Environmental changes promote larger species of Odonata (Insecta) in Amazonian streams. Ecol. Ind..

[38] Myers N, Mittermeier RA, Mittermeier CG, Da Fonseca GA, Kent J (2000). Biodiversity hotspots for conservation priorities. Nature.

[39] Rodrigues ME, Oliveira FR, Quintero JMO, Castro PJC, Sousa DC, De Marco P (2016). Nonlinear responses in damselfly community along a gradient of habitat loss in a savanna landscape. Biol. Conserv..

[40] Peel MC, Finlayson BL, McMahon TA (2007). Updated world map of the Köppen–Geiger climate classification. Hydrol. Earth Syst. Sci. Discuss..

[41] Brasil LS, Giehl NFDS, Almeida SM, Valadão MBX, dos Santos JO, Pinto NS, Batista JD (2014). Does the damming of streams in the southern Amazon basin affect dragonfly and damselfly assemblages (Odonata: Insecta)? A preliminary study. Int. J. Odonatol..

[42] Lencioni FAA (2005). The Damselflies of Brazil: An Illustrated Guide the Non Coenagrionidae Families.

[43] Lencioni FAA (2006). The Damselflies of Brazil: An Illustrated Guide—Coenagrionidae.

[44] Garrison N, Ellenrieder JAL (2010). Louton Damselfly Genera of the New World: An Illustrated and Annotated Key to the Zygoptera University Press.

[45] Frissell CR, Liss WJ, Warren CE, Hurley MD (1986). A hierarchical framework for stream habitat classification: Viewing streams in a watershed context. Environ. Manag..

[46] Espírito-Santo HMV, Magnusson WE, Zuanon J, Mendonça FP, Landeiro VL (2009). Seasonal variation in the composition of fish assemblages in small Amazonian forest streams: Evidence for predictable changes. Freshw. Biol..

[47] Uieda VS, Castro RMC (1999). Coleta e fixação de peixes de riachos: Ecologia de peixes de riacho.

[48] Planquette P, Keith P, Bail PYL (1996). Atlas des poissons d'eau douce de Guyane.

[49] Albert JS. *Species Diversity and Phylogenetic Systematics of American Knifefishes (Gymnotiformes**, **Teleostei)*. (Miscellaneous Publications of the Museum of Zoology of the University of Michigan, Michigan, 2001).

[50] Kaufmann PR, Levine P, Robison EG, Seeliger C, Peck DV (1999). Quantifying Physical Habitat in Wadeable Streams.

[51] Peck DV, Herlihy AT, Hill BH, Hughes RM, Kaufmann PR, Klemm DJ, Cappaert MR (2006). Invironmental Monitoring and Assessment Program-Surface Waters: Western Pilot Study Field Operations Manual for Wadeable Streams.

[52] De Marco P, Nobrega CC (2018). Evaluating collinearity effects on species distribution models: An approach based on virtual species simulation. PLoS ONE.

[53] Legendre P (1993). Spatial autocorrelation: Trouble or new paradigm?. Ecology.

