# Supplementary material for: The importance of common and the irrelevance of rare species for partition the variation of community matrix: implications for sampling and conservation
Source: Sci Rep. 2020 Nov 13;10:19777. doi: 10.1038/s41598-020-76833-5 (PMC7666184; doi:10.1038/s41598-020-76833-5)
Supplement: Supplementary file 1 — Supplementary Information. [file 41598_2020_76833_MOESM1_ESM.docx]

**THE IMPORTANCE OF COMMON AND THE IRRELEVANCE OF RARE SPECIES FOR PARTITION THE VARIATION OF COMMUNITY MATRIX: IMPLICATIONS FOR SAMPLING AND CONSERVATION**

Leandro Schlemmer Brasil ^1, 2^ *, Thiago Bernardi Vieira ^3^, André Felipe Alves Andrade ^4^, Rafael Costa Bastos ^2, 3^, Luciano Fogaça de Assis Montag ^1, 2, 3^ Leandro Juen ^1, 2, 3^

^1^ Programa de Pós-Graduação em Zoologia, Universidade Federal do Pará, Belém, Pará, Brasil.

^2^ Laboratório de Ecologia e Conservação, Universidade Federal do Pará, Belém, Pará, Brasil.

^3^ Programa de Pós-Graduação em Ecologia e Conservação, Universidade Federal do Pará, Belém, Pará, Brasil.

^4^ Theory, Metacommunity and Landscape Ecology Lab, Universidade Federal de Goias, Goiânia, Goias, Brasil.

Odonata

| **Species** | **N** | **Species** | **N** | **Species** | **N** | **Species** | **N** |
| --- | --- | --- | --- | --- | --- | --- | --- |
| *Acanthagrion adustum* | 6 | *Argia reclusa* | 5 | *Epipleoneura pereirai* | 1 | *Mnesarete smaragdina* | 36 |
| *Acanthagrion aepiolum* | 2 | *Argia smithiana* | 1 | *Epipleoneura solitaria* | 1 | *Mnesarete williamsoni* | 61 |
| *Acanthagrion apicale* | 2 | *Argia subapicalis* | 1 | *Epipleoneura westfalli* | 13 | *Neoneura bilinearis* | 2 |
| *Acanthagrion ascendens* | 1 | *Argia thespis* | 36 | *Heliocharis amazona* | 2 | *Neoneura fulvicollis* | 7 |
| *Acanthagrion kennedii* | 4 | *Argia tinctipennis* | 68 | *Hetaerina auripennis* | 8 | *Neoneura gaida* | 125 |
| *Acanthagrion rubrifrons* | 1 | *Argia tupi* | 5 | *Hetaerina indeprensa* | 3 | *Neoneura luzmarina* | 9 |
| *Acanthallagma luteum* | 1 | *Chalcolpterix radians* | 43 | *Hetaerina laesa* | 2 | *Neoneura rubriventris* | 10 |
| *Argia chapadae* | 18 | *Chalcopteryx rutilans* | 98 | *Hetaerina sanguinea* | 1 | *Oxystigma petiolatum* | 9 |
| *Argia collata* | 16 | *Dicterias atrosanguinea* | 10 | *Heteragrion aurantiacum* | 15 | *Perilestes attenuatus* | 1 |
| *Argia dives* | 2 | *Epipleoneura capilliformis* | 17 | *Heteragrion ictericum* | 54 | *Perilestes kahli* | 4 |
| *Argia euphorbia* | 2 | *Epipleoneura fuscaenea* | 6 | *Heteragrion icterops* | 2 | *Phasmoneira exígua* | 14 |
| *Argia fumigata* | 8 | *Epipleoneura haroldoi* | 14 | *Heteragrion silvarum* | 137 | *Phoenicagrion flammeum* | 11 |
| *Argia hasemani* | 22 | *Epipleoneura kaxuriana* | 17 | *Mecistogaster amalia* | 1 | *Protoneura tenuis* | 26 |
| *Argia indicatrix* | 28 | *Epipleoneura machadoi* | 1 | *Metaleptobasis diceras* | 2 | *Psaironeura tenuissima* | 67 |
| *Argia infumata* | 103 | *Epipleoneura metallica* | 68 | *Mnesarete aenea* | 102 | *Telebasis sanguinalis* | 5 |
| *Argia oculata* | 2 | *Epipleoneura ocuene* | 1 | *Mnesarete cupraea* | 15 | *Tigriagrion aurantinigrum* | 8 |

Peixes

| **Fish** | **N** | **Fish** | **N** | **Fish** | **N** | **Fish** | **N** | **Fish** | **N** |
| --- | --- | --- | --- | --- | --- | --- | --- | --- | --- |
| Aca_cat | 1 | Car_str | 160 | Gym_gca | 21 | Itu_sp. | 21 | Par_sp. | 28 |
| Aeq_tet | 553 | Cha_eth | 71 | Gym_gco | 315 | Itu_ama | 251 | Phe_pec | 1 |
| Amm_ele | 6 | Cha_sp. | 4 | Gym_gpa | 149 | Kno_vic | 8 | Phr_cis | 2 |
| Ana_uro | 872 | Cha_zeb | 1 | Gym_sp. | 8 | Lai_str | 496 | Phy_ana | 49 |
| Api_aga | 559 | Cop_arn | 2887 | Hel_mar | 910 | Mas_aso | 46 | Pim_sp. | 17 |
| Api_reg | 3539 | Cor_mel | 3 | Hem_bel | 892 | Meg_pic | 1 | Pot_eig | 1 |
| Ast_bim | 12 | Cre_pun | 3 | Hem_gei | 1 | Meg_tho | 9 | Pot_has | 1320 |
| Bar_ste | 3 | Cre_ret | 5 | Hem_rho | 1 | Mel_dis | 6 | Pyr_bre | 1564 |
| Bat_ran | 7 | Cre_sax | 176 | Hem_rod | 508 | Mic_wei | 6779 | Rha_que | 9 |
| Bra_bee | 226 | Cre_spi | 445 | Hem_sp. | 86 | Mic_bil | 119 | Rha_sp. | 2 |
| Bra_bre | 114 | Cur_cry | 39 | Hem_oce | 418 | Moe_com | 21 | Rha_mue | 19 |
| Bra_bul | 78 | Den_epa | 19 | Her_sp. | 1 | Moe_col | 9 | Rin_has | 12 |
| Bra_sp.1 | 50 | Den_sp. | 1 | Hop_uni | 1 | Mon_oli | 22 | Rin_sp. | 3 |
| Bra_sp.2 | 262 | Eig_tri | 10 | Hop_cur | 2 | Mon_pol | 3 | Sat_jur | 8 |
| Bra_sp.3 | 16 | Eig_sp. | 3 | Hop_mal | 81 | Nan_tae | 506 | Ser_sp. | 3 |
| Bra_sp.4 | 8 | Ery_ery | 704 | Hyp_ben | 12 | Nan_bec | 11 | Ste_ele | 23 |
| Bry_cau | 8 | Far_ama | 72 | Hyp_het | 11680 | Nan_equ | 10 | Ste_ama | 1 |
| Bry_sp. | 26 | Gla_con | 316 | Hyp_lep | 141 | Nan_nit | 108 | Ste_mac | 6 |
| Bun_cor | 48 | Gna_ste | 11 | Hyp_tem | 2 | Nan_tri | 257 | Tet_bar | 3 |
| Cal_cal | 12 | Gym_pet | 824 | Igu_rac | 1473 | Oto_mur | 58 | Tet_wal | 1 |
